# Prevalence and risk factors for low back pain among professional cooks working in school lunch services

**DOI:** 10.1186/1471-2458-7-171

**Published:** 2007-07-24

**Authors:** Miwako Nagasu, Kazuhiro Sakai, Akiyoshi Ito, Shigeru Tomita, Yoshiomi Temmyo, Mitsuo Ueno, Shigeji Miyagi

**Affiliations:** 1 Department of Health Sciences, Kagawa Nutrition University, Saitama, Japan; 2 The Institute for Science of Labour, Kanagawa, Japan; 3 School of Health Sciences, University of Occupational and Environmental Health, Kitakyushu, Japan; 4 School of Medicine, Dokkyo Medical University, Tochigi, Japan; 5 Minatomachi Medical Center, Kanagawa, Japan; 6 Institute of Occupational Safety and Health, All-Japan Prefectural and Municipal Workers' Union, Tokyo, Japan

## Abstract

**Background:**

The prevalence of self-reported low back pain among professional cooks was estimated to examine the effects of daily life conditions, job-related factors, and psychological factors on this disorder.

**Methods:**

Data was collected using a mailed self-administered questionnaire.

**Results:**

Of 7100 cooks, 5835 (82%) replied to the questionnaire, including 1010 men and 4825 women. The mean age was 41.4 for men and 47.5 for women. The prevalence of low back pain during a 1-month period was 72.2% among men and 74.7% among women, with no significant differences between groups. By logistic regression analyses, factors significantly associated with the prevalence of low back pain in 1 month were female gender (prevalence ratio [PR] 1.32; 95% CI, 1.03–1.68), current smoking (PR 1.57; 95% CI, 1.24–1.98), and past smoking (PR 1.35; 95% CI, 1.01–1.79). As for job-related factors, the number of cooked lunches per person (PR 1.28; 95% CI, 1.05–1.56), breaks in the morning session (PR 1.33; 95% CI, 1.13–1.56), kitchen environment (PR 1.09; 95%, CI, 1.03–1.15), and height of cooking equipment (PR 1.13; 95% CI, 1.08–1.19) were associated with the prevalence of low back pain. As for psychological factors, job satisfaction (PR 1.22; 95% CI, 1.03–1.45), stress at work (PR 1.68; 95% CI, 1.42–1.99), financial constraints (PR 1.23; 95% CI, 1.03–1.47), health-related stress (PR 1.31; 95% CI, 1.08–1.59) and worries about the future (PR 1.24; 95% CI, 1.01–1.52) were similarly associated.

**Conclusion:**

Daily life conditions, job-related factors, and psychological factors are associated with the occurrence of low back pain. It is important to take comprehensive preventive measures to address a range of work and life conditions that can be improved to decrease the incidence of low back pain for professional cooks.

## Background

Low back pain (LBP) is the most frequently reported work-related disorder in Japan [1]. Various studies [2,3] have reported that among working populations, the rate of LBP due to heavy workload during the previous day [4], week, month [5,6], or year [7-10], or during the lifetime [4,9,11] varies between 5.6% and 84.1% [12].

LBP is a common disorder among cooks working in school lunch services in Japan [13-17]. Several studies have reported that the prevalence of back disorders during a specific period of employment ranged from 26.4% and 55.3% [14,16,18-20]. The importance of studying risk factors that influence work-related disorders and the occurrence of LBP among the working population [21] is thus emphasized. Some studies report that female gender [2,6,9,11,22-25] or higher age [9,26] increase the risk of LBP, whereas other studies report no association between these factors [6,9].

Recent studies have reported that psychological factors such as job satisfaction [27] and job strain [5,27] also may be risk factors for LBP. However, evidence for an association between psychological factors and LBP among cooks has not been studied in detail. The above-mentioned studies have found that demographic characteristics, daily life conditions, job-related factors, and psychological factors may influence the occurrence of LBP.

These studies indicate the need to take into account multiple work and life factors that can be changed to help reduce the prevalence of LBP, including daily life conditions (lifestyle and dietary habits), job-related factors (work activities and workplace environment), and psychological factors.

There have been reported interactions between the above-mentioned factors. However, many earlier studies did not adjust for potential confounding variables. Therefore studies evaluating the risk factors associated with LBP are inconclusive. There is a lack of well-designed studies on the prevalence and risk factors associated with LBP among professional cooks, particularly those working in school lunch services, as they are exposed to high work loads [13-17]. Therefore, we carried out this study on a nationally representative sample of cooks. The objectives of the study were (1) to estimate the prevalence of self-reported rates of LBP and (2) to identify factors that could influence the occurrence of LBP among cooks. Particular attention was paid to the physical kitchen environment as a potential cause of LBP. Attention was also paid to the links between LBP and psychological aspects of the work environment.

## Methods

We conducted a cross-sectional survey using a mailed self-administered questionnaire that investigated the prevalence of self-reported LBP and its association with various risk factors among professional cooks.

### Study setting and participants

Participants included professional cooks working in school lunch services of the elementary schools of Japan. According to investigations, school lunches were provided at elementary schools all over Japan to about 7.19 million children in 2002 [28]. A total of 46,213 full-time cooks were working in the school lunch services in elementary schools and central kitchen systems in Japan during this period [29].

The principal task of these cooks was to provide meals for lunch for all pupils 5 days a week, year round, excluding seasonal vacation periods. There are two kinds of school lunch services in Japan. One of them consists of individual school kitchens in which lunch services are provided only for a particular school. The second type consists of services provided by medium kitchens and large kitchens, each of which delivers lunch service in large quantities to many schools.

The study sample was selected through the head office of the All-Japan Prefectural and Municipal Workers Union. There are a total of 47 prefectures in Japan. All prefectures and municipalities were included in our study to obtain proper representation of all of Japan. The union leaders maintained a list of the cooks working in school lunch services. A total of 500 cooks from 47 prefectures were selected. Similarly 200 cooks from each of 10 prefectures were selected (total 2000), 300 cooks from each of five prefectures (total 1500), and 100 cooks from each of 31 prefectures (total 3100) were randomly selected from a list of cooks. Thus a total of 7100 cooks were selected for this study.

### Questionnaire

A mailed self-administered questionnaire was used to collect information about LBP and demographic characteristics, daily life conditions, job-related factors, and psychological factors. Prevalence of work-related disorders was ascertained by questionnaires used in previous studies on health hazards among cooks providing school lunch services [19,20]. This enabled us to compare the present study with previous ones. The questionnaire contained information about demographic factors such as age and gender. Daily life factors included information about lifestyle such as smoking, physical activities, and sleeping and eating habits. Information about job-related factors included work activities and conditions in the kitchens. Psychological factors included job satisfaction, stress at work, family-related stress, financial constraints, health-related problems, and worries about the future. Symptoms related to LBP, that is, episodes of pain, stiffness, or discomfort in the low back that were experienced during the previous 1 month and included in the questionnaire were adapted from a Japanese edition of a questionnaire used in previous studies [19,20].

### Data collection

A cross-sectional survey was carried out from September 2003 to February 2004. This study was supported by the All-Japan Prefectural and Municipal Workers Union. A questionnaire was mailed to 7100 professional cooks through the branches of the union located all over Japan. The questionnaire form was enclosed in an envelope that also contained an informed consent form. The anonymity and confidentiality of the information to be provided was detailed in the consent form. The participants were also free to refuse to respond to the questionnaire. The completed questionnaire forms were mailed back to the investigators.

For the purpose of this study, low back pain was defined as an experience of an episode or episodes of pain, stiffness, or discomfort of the low back anytime during the previous 1 month. The prevalence rate of LBP refers to the number of subjects who had reported at least period of LBP during the previous month.

### Data analysis

The statistical analysis was carried out using the SPSS (12.0J Base System) computer package. The prevalence rates and their 95% confidence intervals (CI) were calculated. Descriptive statistics were calculated for continuous variables. For continuous variables, the student t test was used to test the significance of observed differences. Observed differences between frequencies of categorical variables were tested using the chi square test.

We investigated the adjusted prevalence ratios (PR) and 95% CIs of LBP by logistic regression analysis according to the following groups: gender, age, smoking habits, physical activity, sleeping habits, eating habits, work activities, environmental conditions in the kitchen, such as the kitchen environment score and the working height of cooking equipment score, job satisfaction, stress at work, family-related stress, financial constraints, health-related problems, and worries about the future.

The kitchen environment score and the working height of cooking equipment score were determined as follows: The kitchen environment score was estimated as the sum total of points indicating "no rest room," "wet floor system," "poor state of drainage," "presence of slippery places," "bumps and obstacles on the floor," and "noisy surroundings." The working height of cooking equipment score was estimated as the number of different categories of cooking equipment having inappropriate heights, including counter-tables, kitchen sinks, cauldrons, vegetable cutters and peelers, feeding and finishing parts of tableware washing machines, and hot wind tableware dryers.

A generalized log linear model with binominal distribution was used to present associations between risk factors and LBP. PRs were estimatedas a measure of association. PRs were used because they are considered to be a better approximation of relative risk than odds ratio if the disease prevalence is high. Age was considered a potential confounding variable. Age was included in each logistic regression model, regardless of its association with LBP. A series of logistic regression models were used to identify the determinants of LBP. All self-reported risk factors were included in the regression models. PRs, 95% CIs, and p values were calculated for each variable. A p value of 0.05 was considered statistically significant.

## Results

Of 7100 cooks to whom the questionnaire was sent, 6,365 (90%) returned the completed questionnaire forms by mail. Information about age and gender of respondents or other important information was incomplete on 530 forms. These replies were not included in the final analysis. Therefore 5835 replies were analyzed, for an overall response rate of 82%.

Participants included 1010 men (17.3%) and 4825 women (82.7%). The mean ± SD age of men (41.4 ± 9.8 years) was significantly lower than that of women (47.5 ± 9.1 years) (p < 0.001).

The overall 1-month prevalence of self-reported LBP was 74.3% among all the subjects. The prevalence of LBP among men and women was 72.2% and 74.7%, respectively (Table 1). Differences by gender were not statistically significant. Differences in prevalence rates of LBP across different age groups were not statistically significant among men or among women.

**Table 1:** Demographic characteristics of subjects

	LBP in 1 month				
	
	Total	Prevalence (%)	Test^1)^	PR non-adj ^2)^	95% CI
**Gender**					
Male	998	72.2		1	
Female	4781	74.7	n.s	1.13	0.97–1.32
**Age**					
≦39	1400	75.4		1	
40 ~ 49	1764	74.8	n.s	0.97	0.82–1.14
50≦	2615	73.3		0.90	0.78–1.05
**Gender and age**					
**Male**					
≦39	428	73.8		1	
40 ~ 49	337	73.0	n.s	0.96	0.69–1.32
50≦	233	68.2		0.76	0.54–1.08
**Female**					
≦39	972	76.0		1	
40 ~ 49	1427	75.2	n.s	0.96	0.79–1.16
50≦	2382	73.8		0.90	0.75–1.06

1) Chi-square test.

2) Prevalence ratio was not adjusted.

We investigated the following risk factors among men and women: age, smoking habits, physical activities, sleeping habits, eating habits, work activities, workplace environment conditions in the kitchen, job satisfaction, stress at work, family-related stress, financial constraints, health-related problems, and worries about the future. Tables 2, 3, 4 show prevalence rates of LBP according to the risk factors studied and also age-adjusted prevalence ratios of LBP stratified according to gender. As shown in Table 2, LBP was associated with daily life conditions such as smoking, physical activities, sleeping hours, and dietary habits among both men and women.

**Table 2:** Prevalence of self-reported low back pain and the associations with selected daily life conditions

	**Male**					**Female**				
		
	Total	Prevalence(%)	Pvalue^1)^	PR adj ^2)^	95% CI	Total	Prevalence(%)	Pvalue^1)^	PR adj ^2)^	95% CI
Smoking										
Never	260	65.4		1		3812	73.3		1	
Ex-smokers	199	72.4	*	1.41	0.94–2.11	305	78.4	**	1.31	0.98–1.73
Current smokers	523	75.1		**1.59**	**1.15–2.02**	598	81.6		**1.61**	**1.29–2.00**
Physical activity										
Yes, regularly	280	70.7		1		771	70.8		1	
No	718	72.8	n.s	1.15	0.84–1.56	4010	75.4	**	**1.27**	**1.07–1.50**
Sleeping hours										
≦6 hours	553	71.6		1		3102	76.5		1	
7 hours≦	428	72.7	n.s	0.92	0.69–1.22	1632	71.4	**	**1.30**	**1.14–1.49**
Having meals regularly										
Regularly	348	67.8		1		1949	73.5		1	
Irregularly	557	75.2	*	**1.43**	**1.06–1.92**	2636	75.5	n.s	1.11	0.97–1.26

1) Chi-square test was conducted for each item among men and among women. *p < 0.05, **p < 0.01.

2) Prevalence ratio was adjusted for age group.

**Table 3:** Prevalence of self-reported low back pain and the associations with selected job-related factors

	**Male**					**Female**				
		
	Total	Prevalence(%)	Pvalue^1)^	PR adj ^2)^	95% CI	Total	Prevalence(%)	Pvalue^1)^	PR adj ^2)^	95% CI
**Work activities**										
Number of cooked lunches per cook										
1–149 meals	412	69.7		1		2457	71.2		1	
150–199 meals	243	74.9	n.s	1.30	0.91–1.86	1319	79.1	**	**1.53**	**1.30–1.79**
200 meals≦	340	73.2		1.19	0.87–1.60	985	77.5		**1.39**	**1.17–1.65**
Breaks in morning session										
Yes	549	68.9		1		2122	70.2		1	
No	426	77.0	**	**1.50**	**1.13–2.01**	2536	78.4	**	**1.54**	**1.34–1.75**
Type of workplace										
Small kitchen	314	66.6		1		3184	73.4		1	
Medium kitchen (<1500 meals)	78	70.5	*	1.20	0.70–2.06	355	75.2	*	1.10	0.85–1.42
Large kitchen (1500 meals≦)	606	75.4		**1.54**	**1.14–2.08**	1242	77.8		**1.27**	**1.08–1.60**
**Workplace environment factors**										
**Kitchen environment**										
State of drainage										
Good	304	67.1		1		1760	71.5		1	
Poor	684	74.6	*	**1.43**	**1.06–1.92**	2907	76.5	**	**1.29**	**1.23–1.46**
Slippery places on the floor										
No	340	62.6		1		2267	69.9		1	
Yes	637	77.7	**	**2.05**	**1.53–2.74**	2399	78.9	**	**1.60**	**1.40–1.83**
Bumps on the floor										
No	598	68.7		1		2872	72.1		1	
Yes	377	78.2	**	**1.62**	**1.02–2.19**	1810	78.4	**	**1.39**	**1.22–1.61**
Obstacles on the floor										
No	565	69.0		1		3063	73.5		1	
Yes	412	77.2	**	**1.51**	**1.13–2.02**	1599	77.0	*	**1.20**	**1.04–1.38**
Noise level										
Quiet	583	69.5		1		2438	71.1		1	
Noisy	404	76.0	*	**1.41**	**1.06–1.88**	2249	78.6	**	**1.49**	**1.31–1.71**
**Height of cooking equipment**										
Counter-tables										
Fit	568	68.0		1		3467	72.3		1	
High or low	420	78.1	**	**1.66**	**1.24–2.24**	1242	80.9	**	**1.62**	**1.38–1.90**
Kitchen sinks										
Fit	392	67.3		1		2432	68.5		1	
High or low	603	75.5	**	**1.45**	**1.09–1.93**	2274	81.2	**	**2.00**	**1.74–2.29**
Cauldrons										
Fit	772	68.9		1		3717	73.0		1	
High or low	209	85.6	**	**2.65**	**1.75–4.03**	950	80.8	**	**1.55**	**1.30–1.85**
Peelers										
Fit	685	70.4		1		3191	72.1		1	
High or low	254	77.6	*	**1.44**	**1.03–2.02**	1189	81.1	**	**1.64**	**1.39–1.94**
Tableware washing machines – feeding										
Fit	776	70.6		1		3329	73.8		1	
High or low	193	79.8	*	**1.60**	**1.09–2.36**	1071	78.7	**	**1.31**	**1.11–1.55**
Tableware washing machines – finishing										
Fit	694	69.2		1		3013	73.1		1	
High or low	271	81.2	**	**1.89**	**1.34–2.67**	1379	79.0	**	**1.38**	**1.19–1.61**

1) Chi-square test was conducted among men and women for each item. *p < 0.05, **p < 0.01.

2) Prevalence ratio was adjusted for age group.

**Table 4:** Prevalence of self-reported low back pain and the associations with psychological factors

	**Male**					**Female**				
		
	Total	Prevalence(%)	Pvalue^1)^	PR adj ^2)^	95% CI	Total	Prevalence(%)	Pvalue^1)^	PR adj ^2)^	95% CI
Job satisfaction										
Satisfied	438	63.9		1		2944	72.1		1	
Dissatisfied	533	78.6	**	**2.08**	**1.57–2.77**	1719	79.4	**	**1.49**	**1.29–1.71**
Stress at work										
Fine	350	58.9		1		1683	64.5		1	
Stressful	647	79.6	**	**2.71**	**2.03–3.61**	3069	80.3	**	**2.23**	**1.95–2.55**
Family-related stress										
Fine	578	67.3		1		2268	70.9		1	
Stressful	411	79.8	**	**1.94**	**1.44–2.62**	2478	78.2	**	**1.47**	**1.23–1.67**
Financial constraints										
Fine	290	62.4		1		1812	68.3		1	
Stressful	707	76.4	**	**1.91**	**1.42–2.57**	2933	78.6	**	**1.71**	**1.50–1.96**
Health-related problems										
Fine	421	63.2		1		1751	66.5		1	
Stressful	567	79.4	**	**2.32**	**1.74–3.09**	3002	79.5	**	**1.97**	**1.73–2.25**
Worries about the future										
Fine	290	62.1		1		1265	65.1		1	
Stressful	700	76.7	**	**2.01**	**1.49–2.69**	3475	78.1	**	**1.91**	**1.66–2.20**

1) Chi-square test was conducted among men and women for each item. *p < 0.05, **p < 0.01.

2) Prevalence ratio was adjusted for age group.

For women, regular physical activity (PR 1.27; 95% CI, 1.07–1.50), the number of hours of sleep (PR 1.30; 95% CI, 1.14–1.49), and the number of cooked lunches per cook (150–199 meals; PR 1.53; 95% CI, 1.30–1.79, 200 meals≦; PR 1.39; 95% CI, 1.17–1.65) were significantly associated with LBP. For men, none of these factors were associated with LBP; however, the irregularity of eating meals (PR 1.43; 95% CI, 1.06–1.92) was significantly associated LBP among men.

For both sexes, current smoking habits (men; PR 1.59; 95% CI, 1.15–2.02, women; PR 1.61; 95% CI, 1.29–2.00), lack of breaks in the morning session (men; PR 1.50; 95% CI, 1.13–2.01, women; PR 1.54; 95% CI, 1.34–1.75), and working in large kitchens (men; PR 1.54; 95% CI, 1.14–2.08, women; PR 1.27; 95% CI, 1.08–1.60) were significantly associated with rates of LBP. Working environment factors significantly associated with rates of LBP for both sexes included noisy surroundings (men; PR 1.41; 95% CI, 1.06–1.88, women; PR 1.49; 95% CI, 1.31–1.71), poor state of drainage (men; PR 1.43; 95% CI, 1.06–1.92, women; PR 1.29; 95% CI, 1.23–1.46), and the presence of slippery places (men; PR 2.05; 95% CI, 1.53–2.74, women; PR 1.60; 95% CI, 1.40–1.83), bumps (men; PR 1.62; 95% CI, 1.02–2.19, women; PR 1.39; 95% CI, 1.22–1.61), and obstacles on the floor (men; PR 1.51; 95% CI, 1.13–2.02, women; PR 1.20; 95% CI, 1.04–1.38).

As for height of cooking equipment, several items with variable working heights were significantly associated with rates of LBP, including counter-tables (men; PR 1.66; 95% CI, 1.24–2.24, women; PR 1.62; 95% CI, 1.38–1.90), kitchen sinks (men; PR 1.45; 95% CI, 1.09–1.93, women; PR 2.00; 95% CI, 1.74–2.29), cauldrons (men; PR 2.65; 95% CI, 1.75–4.03, women; PR 1.55; 95% CI, 1.30–1.85) and peelers (men; PR 1.44; 95% CI, 1.03–2.02, women; PR 1.64; 95% CI, 1.39–1.94), feeding (men; PR 1.60; 95% CI, 1.09–2.36, women; PR 1.31; 95% CI, 1.11–1.55) and finishing (men; PR 1.89; 95% CI, 1.34–2.67, women; PR 1.38; 95% CI, 1.19–1.61) parts of tableware washing machines.

A number of psychological factors were significantly associated with the prevalence of LBP in both sexes, including job satisfaction (men; PR 2.08; 95% CI, 1.57–2.77, women; PR 1.49; 95% CI, 1.29–1.71), stress at work (men; PR 2.71; 95% CI, 2.03–3.61, women; PR 2.23; 95% CI, 1.95–2.55), family-related stress (men; PR 1.94; 95% CI, 1.44–2.62, women; PR 1.47; 95% CI, 1.23–1.67), financial constraints (men; PR 1.91; 95% CI, 1.42–2.57, women; PR 1.71; 95% CI, 1.50–1.96), health-related problems (men; PR 2.32; 95% CI, 1.74–3.09, women; PR 1.97; 95% CI, 1.73–2.25), and worries about the future (men; PR 2.01; 95% CI, 1.49–2.69, women; PR 1.91; 95% CI, 1.66–2.20).

The results of the regression analyses confirmed the significance of the relationship between many of these factors and the prevalence of LBP, as shown in Table 5. Females (PR 1.32; 95% CI, 1.03–1.68) as well as current (PR 1.57; 95% CI, 1.24–1.98) and ex-smokers (PR 1.35; 95% CI, 1.01–1.79) were more likely to suffer from LBP. Among work activities, cooking more lunches per person (PR 1.28; 95% CI, 1.05–1.56) and a lack of breaks in the morning session (PR 1.33; 95% CI, 1.13–1.56) were associated with LBP. In addition to these factors, the scores of kitchen environment (PR 1.09; 95% CI, 1.03–1.15) and the working height of the cooking equipment (PR 1.13; 95% CI, 1.08–1.19) were also associated with LBP. The median (interquartile range) of the kitchen environment score was 2 (1–4). The median (interquartile range) of the working height of cooking equipment score was 2 (1–3). Among the psychological factors, job dissatisfaction (PR 1.22; 95% CI, 1.03–1.45), stress at work (PR 1.68; 95% CI, 1.42–1.99), financial constraints (PR 1.23; 95% CI, 1.03–1.47), health-related problems (PR 1.31; 95% CI, 1.08–1.59), and worries about the future (PR 1.24; 95% CI, 1.01–1.52) were significantly associated with the higher rates of LBP. It was noteworthy that the highest prevalence ratio was found for stress at work.

**Table 5:** Adjusted prevalence ratios of low back pain for each factor

		PR adj^1)^	95% CI
Gender	Female	1.32	1.03–1.68
Smoking	Ex-smokers	1.35	1.01–1.79
	Current smokers	1.57	1.24–1.98
The number of cooked lunches per person	150–199 meals	1.28	1.05–1.56
No breaks in morning session		1.33	1.13–1.56
Kitchen environment score^2)^		1.09	1.03–1.15
The height of cooking equipment score^3)^		1.13	1.08–1.19
Job satisfaction	Dissatisfied	1.22	1.03–1.45
Stress at work	Stressful	1.68	1.42–1.99
Financial constraints	Stressful	1.23	1.03–1.47
Health-related stress	Stressful	1.31	1.08–1.59
Worries about the future	Stressful	1.24	1.01–1.52

1) Prevalence ratios were adjusted for all items. Prevalence ratios referred to the following groups: male, age <39 years, not smoking, doing physical activity, sleeping for less than 7 hours, having meals regularly, 1–149 meals per a cook, small-scale kitchen, having breaks in the morning session, and all psychological factors unstressful.

2) Kitchen conditions score is the sum total of the points such as no rest room, wet floor, poor state of drainage, presence of slippery places, bumps, and obstacles on the floor, and noisy surroundings (1 point each).

3) The height of cooking equipment score is the sum total of the points such as the inappropriate height of counter-tables, kitchen sinks, cauldrons, vegetable cutters and peelers, feeding and finishing parts of tableware washing machines, and hot wind tableware dryers (1 point each).

## Discussion

In this cross-sectional study, the prevalence of self-reported LBP was high among professional cooks working in school lunch services. This high prevalence was noted regardless of age and gender. The questionnaire results indicated that the prevalence of LBP was associated with daily life conditions, job-related factors, and psychological factors. These results are important as the study was conducted in a large sample of both male and female cooks selected from all over Japan, with a good response rate of 82%. This high response rate was possible through the inclusion of an informed consent form and the assurance of confidentiality of the information provided. The results may thus be considered representative of cooks engaged in public school lunch services.

It was of particular interest that 17.3% of our sample was male, considering that the proportion of male cooks in school lunch services is low [16,17]. Previous studies emphasized the importance of considering the health conditions of male and female cooks separately due to differences in their average height [3,21].

One previous study reported that the prevalence of LBP was associated with stature among men [22]. Further studies may be necessary as this association of weight and tall stature with the occurrence of LBP was not reported in other studies [3,25,30]. As male and female cooks in our study shared the same kitchen and cooking equipment, the significant difference in the mean height of male cooks (169.0 ± 6.60 centimeters) and that of female cooks (156.4 ± 5.17 centimeters) was notable. To prevent LBP, it may be worth considering adjusting the working height of equipment in kitchens used for school lunch services to suit the male and female cooks.

### Prevalence of LBP

The 1-month prevalence of LBP was 74.3% among all the subjects. This prevalence rate was higher than that reported in the previous studies [5,20,31]. The observed prevalence of LBP among cooks was higher than that in the general population (35% and 42% among males and females, respectively) [6], staff members working in schools for physically and mentally handicapped children (45%) [5], or physiotherapy students (44%) [31]. The prevalence of LBP among cooks in our study was similar to the 1-month prevalence (81%) reported by a previous study [32]. Kurumatani et al. reported that the rates of complaints related to the cervicobrachial area and low back were significantly higher in female cooks than in housewives of similar age [32]. Other previous studies also reported that the 1-month prevalence of LBP was 68% among cooks working in large kitchens [19] and 67% among cooks younger than 35 years [16]. The high prevalence of LBP among cooks reported by these studies may warrant the need to undertake preventive measures for improving health and work efficiency of professional cooks.

### Demographic characteristics

According to our logistic regression analyses, LBP was associated with several risk factors. It was noteworthy that LBP was associated with gender after adjustment for all factors. The association of LBP with gender has also been reported by other studies [3,24,26]. LBP has also been shown to be more common among women than men in some occupations, such as white collar workers [22,23,33], whereas it is more frequent for men than for women among peasant farmers [26]. We confirmed that among the professional cooks in Japan, the risk of LBP was higher among women than men.

In this study, age was not associated with the prevalence of LBP among cooks. Some studies have reported that the prevalence of LBP increases with age among farmers [26] and hospital staff [24]. Other studies have reported no associated between age and LBP [3].

### Daily life conditions

We found a significant relation between smoking habits and the prevalence of LBP among cooks. Our study showed that cooks who were current and ex-smokers had a higher prevalence rate of LBP than those who had never smoked. After adjusting for all risk factors, current smokers had PR for LBP that was 1.5 times higher than that of non-smokers. Previous studies have reported that the incidence of LBP was higher among adolescents and working groups who had ever smoked [24]. The evidence for the association of smoking with LBP was also reported in studies on smoking and decreased bone mineral density in the lumber spine [34]. Gilgil et al. [9] reported that individuals who smoked 21 or more cigarettes per day for 15 years were more likely to suffer from LBP. The results of our study confirm the association of the occurrence of LBP with smoking habits.

Beija et al. reported that playing sports had a protective effect against LBP [24]. In our study, cooks who participated in regular physical activity reported a lower prevalence of LBP. Available literature about the association of LBP with regular sports/physical activity is inconclusive. Further research is needed to prove the effectiveness of sports/physical activity for preventing LBP among the working population.

After adjustment for age, the average duration of sleep was associated with LBP. Women cooks who slept for 7 hours or more per day reported a lower prevalence of LBP than women who slept for less than 7 hours. Generally, women slept for a shorter duration than men. This may be because women have additional responsibilities of housekeeping and childcare. Some studies indicate that housekeeping work and childcare could increase the risk of LBP among women [35-37].

### Job-related factors

In our study, there was a clear association between job-related factors and LBP among cooks. Previous studies dealing with professional cooks also reported that job-related factors such as work activities, workplace conditions in the kitchen, and inappropriate cooking equipment increased the risk of LBP [16,19,20,32,38].

Our study found that physical kitchen environment was variously associated with the prevalence of LBP. For example, cooks who took breaks in the morning session reported LBP less frequently than those who did not. A previous study reported that more frequent tasks involving materials handling, including working with heavy loads, during the morning hours than in the afternoon [14]. It has been suggested that introducing a period of rest in the morning session may have a positive influence on recovery from fatigue and reduction of symptoms such as pain and stiffness, especially in the back, neck and upper limbs among cooks [14,16]. It would be of interest to study the timing and duration of the break period during working hours.

It was found that the number of lunches cooked per person was associated with the occurrence of LBP. This was similar to results of a previous study in which the number of lunches cooked per person was associated with the rate of cervicobrachial complaints and LBP [32]. These findings suggest that the numbers of lunches cooked per person may relate to an increased workload, leading to a higher rate of LBP. A previous study [19] reported that the number of lunches cooked in large-scale kitchens was more than that in small-scale kitchens. Cooks who worked in large-scale kitchens were frequently assigned to carry of heavy objects. These heavier loads may relate to the more frequent occurrence of LBP and cervicobrachial symptoms [17,22]. These symptoms were less commonly reported by the cooks working in small-scale kitchens. However, in the multivariate model, cooks working in the biggest-scale kitchens were not shown to have an increased incidence of LBP.

The kitchen condition score in our study, which included the items such as noisy surroundings, poor state of drainage, and the presence of slippery places, bumps, and obstacles on the floor was associated LBP. In terms of noise in the working environment, a previous study reported that there were places in kitchens where 85 dB (A) was exceeded [14]. Measures against noise are thus necessary in kitchens.

In our study, working in kitchens with obstacles, including height differences, bumps, and slippery places was associated with a higher prevalence of LBP. This may be because pushcarts designed to reduce the need for lifting heavy loads cannot be used in such kitchens. A previous study [16] reported that handling of heavy materials affected the health of workers. A similar association was found between frequent manual handling of materials [17,22] such as ingredients, tableware, and the prevalence of LBP among cooks [21,39]. These findings indicate the need to improve conditions of materials handling to help prevent LBP among cooks.

We found that the working height of cooking equipment, such as counter-tables, kitchen sinks, cauldrons, vegetable cutters, peelers, tableware washing machines, and tableware dryers was associated with the occurrence of LBP. These results were similar to those reported earlier that the risk of LBP increased if the height of cooking equipment did not suit the appropriate working height of the cooks [16,32]. According to Koda et al. [14], the workload of cooks was larger if they maintained uncomfortable standing postures, such as forward bending [16]. Poor working postures are thus known to increase the rate of LBP [40]. Training in working postures [40] and improvement of the working height of all types of cooking equipment may help reduce the rate of LBP.

### Psychological factors

Psychological factors related to work, such as high work stress and low job satisfaction, have been linked with LBP in previous studies [27]. Nevertheless, the relationship between psychological factors and LBP among cooks has not been explored in detail. Our study confirmed a close association between psychological factors such as job satisfaction, stress at work, financial constraints, health-related problems, and worries about the future and the occurrence of LBP among cooks. In our study, higher rates of LBP were observed among the cooks who reported a higher level of stress. In a previous study, workers exposed to high stress at home and at work also suffered a significantly higher rate of LBP [40]. The need for training cooks to manage stress at the workplace and at home should be emphasized.

It may be vital to examine the health status of cooks in relation to their workplace conditions because it is reported that these conditions could cause not only LBP but also complaints in the neck, shoulders, and arms [18]. LBP is known to be a major reason for the loss of work days and therefore decreased productivity [12]. Many studies have confirmed that work-related injuries, disorders, and diseases can be prevented by proper risk assessment and employee training [41-44]. A need is suggested for conducting a longitudinal study to confirm the relationships observed in this study. Information on these relationships would be useful for developing training programs to prevent LBP among cooks.

## Conclusion

The self-reported 1-month prevalence of LBP was high among professional cooks. This high prevalence was significantly associated with daily life conditions, job-related factors, and psychological factors. These results suggest the importance of taking comprehensive preventive measures. For reducing the prevalence of low back pain among cooks, it is generally necessary to address a range of work and life factors. Our results suggest the particular importance of improving the height of kitchen equipment, the physical environment of the kitchen, and other conditions relating to psychosocial aspects. It is useful to develop training programs for school managers and cooks aimed at the prevention of work-related disorders such as LBP.

## Abbreviations

LBP: Low back pain

PRs: Prevalence Ratios

## Competing interests

The author(s) declare that they have no competing interests.

## Authors' contributions

MN: was the principal investigator, participated in the design and protocol preparation, participated in the data collection, data analysis and interpretation of results, and wrote and revised initial drafts of the manuscript.

KS: assisted in design and protocol preparation, and made a substantial contribution toward analysis, drafting the manuscript, and correcting the initial and final drafts of the manuscript.

AI: assisted in design and protocol preparation, made a substantial contribution to the analysis, drafting of manuscript and correcting the initial and final drafts of the manuscript.

ST: provided assistance with the analysis and manuscript preparation.

YT: contributed to the protocol, and helped with the design of the study and manuscript preparation.

MU: helped with the design, protocol, and data collection.

SM: conceived the study, set up the design, guided and participated in the analysis, and revised the final manuscript.

All authors read and approved the final manuscript for submission for publication.

## Pre-publication history

The pre-publication history for this paper can be accessed here:


